# Supplementary material for: Adaptation and pilot testing of the Early Learning Outcome Measure (ELOM) (4&5) Years Assessment tool in South African Sign Language
Source: PLOS Glob Public Health. 2025 Sep 4;5(9):e0005117. doi: 10.1371/journal.pgph.0005117 (PMC12410763; doi:10.1371/journal.pgph.0005117)
Supplement: S1 Table — (DOCX) [file pgph.0005117.s001.docx]

**S1 Table. ELOM 4&5 scoring and cut offs by domain and age**

| **Age and performance band** | | **Domain** | | | | | **Total** |
| --- | --- | --- | --- | --- | --- | --- | --- |
|  |  | GMD (Max score 20) | FMC & VMI (Max score 20) | ENM (Max score 20) | CEF (Max score 20) | ELL (Max Score 20) | Total ELOM (Max score 100) |
| **50-59 mths** | On Track | 8.6 - 20 | 12.32 - 20 | 9.33 - 20 | 7.37 - 20 | 10.26 - 20 | 46.32 -100 |
|  | Falling Behind | 5.41 – 8.59 | 9.71 – 12.31 | 6.35 – 9.32 | 4.08 – 7.16 | 6.54 – 10.25 | 36.02 - 46.31 |
|  | Falling Far Behind | 0 – 5.4 | 0 – 9.70 | 0 – 6.34 | 0 – 4.07 | 0 - 6.53 | 0 - 36.01 |
| **60–69 mths** | On Track | 10.54 - 20 | 14.13 - 20 | 10.24 - 20 | 9.27 - 20 | 11.65 - 20 | 54.38 - 100 |
|  | Falling Behind | 7.22 – 10.53 | 11.47 – 14.12 | 6.91 – 10.23 | 5.82 – 9.26 | 7.98 - 11.64 | 43.24 - 54.37 |
|  | Falling Far Behind | 0 – 7.21 | 0 – 11.46 | 0 – 6.90 | 0 – 5.84 | 0 - 7.97 | 0 - 43.23 |
