# Supplementary material for: Adaptation and pilot testing of the Early Learning Outcome Measure (ELOM) (4&5) Years Assessment tool in South African Sign Language
Source: PLOS Glob Public Health. 2025 Sep 4;5(9):e0005117. doi: 10.1371/journal.pgph.0005117 (PMC12410763; doi:10.1371/journal.pgph.0005117)
Supplement: Attachment — Submitted filename: Responses to reviewers.docx [file pgph.0005117.s004.docx]

**Responses to reviewers’ comments**

We thank the reviewers for their care and also their support of our work. We have uploaded a tracked changes copy and a clean copy as requested. We have addressed the points in the feedback below including those on the copy one reviewer had marked up with changes in the text.

**Please address the reviewers' concerns regarding methodological clarity & language use throughout the manuscript.**

We have paid close attention to the mark up of the pdf manuscript provided by one reviewer and to the list of potential linguistic/grammar errors provided by the other and made changes accordingly as shown in the revised files. Methodological clarity is addressed in specific responses to the comments below.

**Inclusivity in global research questionnaire**

We have completed this and uploaded a copy under supporting information. We have also added the recommended sentence to our methods section: “Additional information regarding the ethical, cultural, and scientific considerations specific to inclusivity in global research is included in the Supporting Information.”

**Please review your reference list to ensure that it is complete and correct.** **If you have cited papers that have been retracted, please include the rationale for doing so in the manuscript text, or remove these references and replace them with relevant current references.** **Any changes to the reference list should be mentioned in the rebuttal letter that accompanies your revised manuscript.** **If you need to cite a retracted article, indicate the article’s retracted status in the References list and also include a citation and full reference for the retraction notice.**

This has been checked. No references have been removed but two have been added.

**The paper is long; trimming/condensing repetitive policy background in the Introduction would improve readability**

We have followed the PLOS guidelines carefully to ensure that only required information has been included and trimmed it considerably before submission to remove extraneous detail. We have carefully looked at any potential repetition in the introduction and policy background as the reviewer pointed this out. We did not find any because we were careful to take the reader through general ECD considerations, to those specific to SA that resulted in policy changes, to how they resulted in the ELOM then how that interfaced or did not with the needs of deaf children’s early development. None of that information in the introduction was repetitive. It was additive to make the case for why this piece of work was required. It is true that some information appears in the abstract also but that is correct given that the abstract will be searched for independently via digital data bases. Also there is some reprise of key points in the discussion that contextualise the findings further but only in so far it is required to make sense of the data.

**Is there other Deaf ECD measurement work in low- and middle-income countries (e.g. Kenya, India) that could position the contribution globally?**

Although we are aware of a range of assessment and measurement work in many countries around the world concerning deaf children’s language development but we are aware of no other psychometric assessment that focusses on Early Childhood Development that is adapted for or includes specifically Deaf children in LMICs. We consulted a new systematic review of the psychometric properties of 117 ECD assessments and none of the 160 papers included involved deaf children specifically because they were not adapted for the different demands that these children might make on the validity of the instruments and their administration. We have included this new reference in our revisions.

**In terms of the technical rigor and methodological detail, the paper presents an excellent outline of the Delphi process, but the final adaptation decisions are said to be presented “in a forthcoming article”; it is just noted that without those details, replication is impossible.**

We have modified our wording of this point in our manuscript to emphasise that this is the topic of a following paper rather than paper that can be referenced in present time.

**In terms of the psychometrics, n = 29 (vs planned 49) is severely under-powered; several domains show α < 0.6 and very wide Cis on test–retest r. The authors note this throughout, but they could consider tempering conclusions further and label findings “preliminary”.**

We have been careful throughout not to over claim the significance of our results but we take the reviewer’s point and have added ‘preliminary’ where suitable on the main text and in the abstract.

**The authors could consider using intraclass correlation coefficients (ICC 2,1) rather than Pearson r for test–retest reliability, as ICC incorporates systematic mean shifts, and consider adding standard error of measurement (SEM) and Bland–Altman plots to visualise agreement.**

Thank you for this suggestion. We chose Pearson r in line with other studies of test/retest reliability that have been undertaken for different language samples of ELOM assessment and because it was the test preferred in the original standardisation. Based on the reviewer’s comment, we did run the alternative ICC approach which we accept gave us a little more granularity but did not materially affect the outcome or conclusions we are drawing from the results. Essentially the same domains were indicated as showing greater reliability than others in line with the original approach to analysis with some minor variation in the strength of that reliability but nothing that was obviously different from our original analysis. On balance therefore we have kept our original approach to analysis as it is valid and is consistent with previous studies thus adding to the body of ELOM work that has used the same approach. Also fundamentally the same conclusion persists which is that the sample is too small and too varied to draw any meaningfully definitive conclusions. We also ran the Bland-Altman plots to visualise agreement which reinforce our previous comments about wide confidence intervals already noted and differences in reliability between domains too. We concluded that the visual representation did not really add anything meaningful to the conclusion already reached. However, when we progress to a bigger sample, we will definitely include the two suggestions of the reviewer in our battery of analyses, because we suspect that with a much bigger sample these approaches will be sensitive to subtleties that would otherwise be missed and the visual representation in particular of the Bland Altmann plots will be useful. It was a helpful exercise to undertake this additional exploration. Thank you.

**The study is essentially a measurement-property study; consider completing a COSMIN checklist and provide as supplementary material.**

The COSMIN checklist is designed to evaluate the methodological quality of studies on measurement properties of health-related patient reported outcomes, commonly termed PROMS (Patient Reported Outcome Measures). More recently the checklist has been expanded to evaluate bias. We did not consider using it because the ELOM-D (4&5) is not a health-related assessment, it is a developmental assessment. Whilst we accept that health plays a role in early childhood development, the instrument itself is not a health-reported outcome measure. We appreciate the idea of using COSMIN but after consideration do not conclude that it is necessary. We accept the reviewer’s point that our work could be viewed as a measurement-property study but not all measurement property studies are patient reported outcome measures (PROMs)

**Also ensure corresponding R code / syntax for scoring is also available.**

We used SPSS as stated for the statistical analysis rather than R. The scoring follows that which was established by the ELOM itself. We have referenced the ELOM Technical Manual where this scoring might be found.

**The interpretation heavily attributes low scores to language deprivation. While plausible, alternative explanations (measurement artefact, cultural unfamiliarity with some tasks, test-environment factors) could be acknowledged. A possible suggestion is to provide a short roadmap for Phase 2 validation.**

We already have offered some alternative explanations for the low scores of the test subjects including whether they are an artefact of the test administration. However, we have strengthened these points by adding a new paragraph about the internal consistency results that includes acknowledgement of the role of underpowered sample in those results as well as pointing to other factors that might be revealed by the intended analysis of the semi-structured interviews of the test administers in due course.

We have indicated already that a greater sample will be required for full validation but have reemphasised this further in the conclusion section.

Specific comments on the text:

**- In abstract percentages oscillate between words and numerals (“Seventy two percent” vs “28 %”). Please standardise.**

We have not made this change as correct written style for a journal article such as this would be to spell out the percentage in full words if it occurs at the start of a sentence but use numbers for percentages otherwise. We have, however, done a thorough check though the rest of the article to ensure this consistency of rule applies.

**- Sentence in l. 55-57 needs reformulation – something is missing**

Word has been added

**- l.64 bracket is missing after ELOM**

Order of acronym and full title have been reversed as this is the first time it is mentioned.

**- not sure if the term “groundbreaking” is appropriate in an academic article**

removed

**- sentence/clause punctuation (specifically comas, semi-colons) missing in places, e.g. l.164, 166, 167, 192, …)**

We have reviewed and changed where necessary.

**- check tense consistency, e.g. l.170 included -> includes**

We have reviewed tense use throughout. When reporting something that occurred in our research process we use the past tense but within that reporting if a designation is ongoing (such as the definition of a term or process) i.e. is present continuous – then in some instances the present tense would be correct. E.g. we evaluated the designations given that X means this and Y means this.

**- what does “(or similar)” mean in l.205**

This was an error and we have removed it

**- quite a bit of repetition on p. 12 (l.202-230); this could be tightened up**

We have tightened up quite a lot in the overview of the article to avoid unnecessary repetition

**- state Delphi consensus threshold (e.g. ≥ 70 % agreement)**

Yes this is added but the nature of the Delphi meant that in the early stages anomalous or single responses could sometimes be significant so we have noted this also.

**- what is a “bespoke workforce”?**

The following description answers that question in that they are SASL users as stated so bespoke for this language group.

**- l.241 be consistent with tense use**

See point above about appropriate tense use and we have checked consistency

**- l.252 reorder clauses**

This has been checked

**- unnecessary to report interpretation of values of Cronbach’s alpha (l.303-304)**

We have removed these.

**- l.339 should this be age of amplification**

Added, thank you.

**- some tables require formatting attention**

We have checked the formatting

**- l.421 has -> had**

Corrected

Further responses based on comments one reviewer put onto the actual pdf of the draft paper

**It should be noted that the internal consistency of the items may reveal either 'unacceptable or poor' largely because of two cogent factors which are (i) the number of items under examination, and (ii) population (n = ?) of data being run for internal consistency. Therefore, it is my personal hypothesis that the 'unacceptable or poor' values displayed n Table 5 are influenced by the two cogent factors.**

We have addressed this more in additions to the revised discussion.

**Does this statement speaks to Deaf children? If yes, can the statement be buttress with the fact that such 'small degree of latitude' is influenced by language difficulties?**

We have reworded this whole paragraph to avoid confusion and accept it was not originally well expressed. We that the reviewer for pointing this out.

**More research to make the justification for the 'small degree in latitude' as earlier written above.**

We have removed the phrase small degree of latitude because it was confusing the main issue which was to do with adaptation of test items rather than adaptation of assessor's instructions and both of these points are made clear in the later discussion of the Delphi. This was just intended as an introductory orientation to the problem and we made it more complex than was required hence our changes now in the revised text.

**Great. However, this statement need to be discussed alongside existing studies on reliability coefficients to affirm the need for large sample size. Although, getting a large sample size for children who are Deaf could be challenging because some are 'hard-to-reach' due to undisclosure of parents or guardians, relatively scattered population of deaf children, parents disbelief and denials....all these factors could be incorporated in the discussion.**

We have reported the factors that affected our recruitment for this study already. We agree looking forward to a phase 2 validation there will be other factors that could be considered but we have confined ourselves to those we investigated when we realised the recruitment was lower than expected specifically in Gauteng.
